# Air, Dermal, and Urinary Metabolite Levels of Backpack and Tractor Sprayers Using the Herbicide Acetochlor in Thailand

**DOI:** 10.3390/toxics11070622

**Published:** 2023-07-18

**Authors:** Nichcha Kallayanatham, Sumate Pengpumkiat, Pornpimol Kongtip, Ritthirong Pundee, Noppanun Nankongnab, Amarin Kongtawelert, Susan R. Woskie

**Affiliations:** 1 Department of Occupational Health and Safety, Faculty of Public Health, Mahidol University, 420/1 Rajvidhi Road, Bangkok 10400, Thailand; nichcha.kal@gmail.com (N.K.); sumate.pen@mahidol.ac.th (S.P.); noppanun.nan@mahidol.ac.th (N.N.); amarin.kon@mahidol.ac.th (A.K.); 2 Nakhonsawan Campus, Mahidol University, Nakhonsawan 60130, Thailand; rtg.pun@gmail.com; 3 Department of Public Health, University of Massachusetts Lowell, Lowell, MA 01854, USA; susan_woskie@uml.edu

**Keywords:** acetochlor, herbicide, breathing zone air sample, dermal patch sample, urinary metabolite, backpack sprayer, tractor sprayer, sugarcane farmer

## Abstract

Acetochlor is a chloroacetanilide selective pre-emergent herbicide used for controlling grass and broadleaf weeds in crops. This study compared the acetochlor exposures of backpack and tractor sprayers and assessed whether dermal or air exposures were more important contributors to the overall body burden as measured by urinary metabolites. Sixty sugarcane farmers in Nakhonsawan province, Thailand participated in the study, and breathing zone air and dermal patch samples were collected during spraying. Urine samples were collected before spraying, at the end of the spraying task, and on the day after spraying. For backpack and tractor sprayers, there was no significant difference in their breathing zone air concentrations, total body dermal samples, or urinary 2-methy-6-methyaniline (EMA) concentrations on the day after spraying. In addition, although most backpack and tractor sprayers wore long pants and long sleeve shirts, they were still exposed to acetochlor, as evidenced by a significant increase in the urinary EMA from before spraying (GM = 11.5 µg/g creatinine) to after spraying (GM = 88.5 µg/g creatinine) to the next day (GM = 111.0 µg/g creatinine). Breathing zone air samples were significantly correlated with those of total body dermal patch samples and with urinary EMA concentrations after spraying. This suggests that both air and dermal exposure contribute to urinary EMA levels.

## 1. Introduction

Agriculture is considered the main occupation for the majority of the population in Thailand, with approximately 47% of the country’s total land area being used for agricultural purposes [1]. However, most farmers are still considered informal workers, meaning they can rarely access workers’ compensation or a government retirement. According to a report in 2022 from the National Statistical Office, 52.5% of informal workers were farmers [2]. It is the largest category of informal work, which makes up 51% of the total Thai workforce. A major hazard for agricultural workers is exposure to hazardous chemicals, including pesticides [2]. According to a 2017 report from the Bureau of Occupational and Environmental Diseases in the Ministry of Public Health, there were 10,312 pesticide poisoning cases, with a rate of 17.12 per 100,000 population [3]. Additionally, the importation of pesticides increased in 2021 compared to 2020, with herbicides comprising the largest volume of pesticides imported into Thailand [4].

Acetochlor (2-chloro-N-(ethoxymethyl)-N-(2-ethyl-6-methylphenyl)-acetamide) is a chloroacetanilide selective pre-emergent herbicide in the chloroacetanilide group used for controlling grass and broadleaf weeds in crops such as corn [5]. Acetochlor was registered in the US in 1994 [6]. Thailand imported about 2661 tons of Acetochlor in 2021 [7] and it is widely used by sugarcane farmers in Thailand, especially in the Nakhonsawan province [8]. 

The US EPA has classified acetochlor as likely to be carcinogenic to humans because it induces nasal tumors in rats at a maximum tolerated dose of 1000 parts per million [9,10]. Previous human studies from North Carolina in 2010 and Iowa in 2011 showed an increased risk of lung, colorectal, and pancreatic cancers, as well as melanoma, among acetochlor-exposed applicators [11]. Previous research in animals has shown that acetochlor may have genetic toxicity and alter thyroid hormone-dependent gene expression in frog tadpoles [12]. In addition, the long-term exposure of rats to acetochlor caused liver and kidney damage and dysfunction of the antioxidant system [13]. In animals, after absorption, acetochlor was also widely distributed in well-perfused organs and showed a low potential for bioaccumulation. Most (66–72%) acetochlor was eliminated in the urine in about 48 h and 12–21% was eliminated in the feces in 48 h [14].

Conventional farmers who use acetochlor choose from several types of available spraying equipment for their agricultural work. Backpack equipment in this study consisted of a manually operated or motorized backpack, which is carried on the back of the operator. The capacity of these sprayers is mostly around 20 L or less [15]. On the other hand, a tractor is a large vehicle that is used for farming and other agricultural activities. Tractors are equipped with mechanized spraying attachments and a maximum of 20 nozzles that can cover a large area. The capacity of these sprayers is 200–400 L [16]. The main difference between a backpack sprayer and a tractor sprayer is the scale of operation. Backpack sprayers are suitable for small-scale operations, while tractors are usually used for large-scale operations [17].

The goal of this study was to compare the exposures of farmers using backpack versus tractor spraying of the herbicide acetochlor and assess whether dermal exposure or air exposure were more important contributors to the overall body burden of farmers.

## 2. Materials and Methods

### 2.1. Study Population and Data Collection

This study was approved by the Ethical Review Committee for Human Research, Faculty of Public Health, Mahidol University (Approval No. MUPH 2015-146, date of approval: (28 August 2015)). From our longitudinal study of conventional farmers (*n* = 213) and organic farmers (*n* = 225), we recruited seventy-eight sugarcane conventional farmers from Khao Thong Subdistrict, Phayuha Khiri District in Nakhonsawan province in Thailand [18]. Informed consent was obtained from all farmers. Data collection for this study was carried out on the recruited conventional sugarcane farmers who sprayed acetochlor during March–May 2017 (*n* = 60). The field staff set an appointment with the farmers for when they planned to spray acetochlor. The type of acetochlor used was 2-chloro-N-ethoxymethyl-6-ethylaceto-o-toluidide 50% *w*/*v* emulsifiable concentrate with a tradename of Razer (in Thai). The backpack sprayers usually mixed 200–4000 mL of acetochlor in 40–200 L of water (concentration range of 5–20 mL/L); the tractor sprayers mixed 1500–8000 mL of acetochlor in 300–1000 L of water (concentration range of 5–8 mL/L). In this study, we selected subjects who sprayed only acetochlor (61.7%) as well as some who sprayed acetochlor mixed with other chemicals, such as paraquat, 2,4-dichlorophenoxyacetic acid, cypermethrin, ametryn, chlorpyrifos, and diuron (38.3%).

Before the spraying day, urine samples were collected at waking in a polyethylene bottle as the first morning void and stored in an insulated ice box, which was held until collection by field staff later in the day. On the spray day, the field staff set up an air sample collection and put the dermal patches on the subjects. The field staff also observed the mixing and spraying and interviewed the subject using a questionnaire. The subject then provided a urine sample at the end of the spraying event. The questionnaire covered demographic information, agricultural activities, history of pesticide mixing and spraying, use of personal protective equipment, and health symptoms after spraying pesticides. Following the day of spraying, the subjects collected their first morning void urine and stored it in an insulated icebox awaiting field staff pickup later in the day. The urine samples were frozen at −20 °C until analysis. 

#### 2.1.1. Personal Breathing Zone Air Sampling

While the farmer was spraying acetochlor, personal air samples were collected according to NIOSH method 5602 using an XAD-2 sorbent tube connected to a glass fiber filter placed in the breathing zone of the farmer [19]. The personal pump was calibrated at 1 L/min before sampling and checked after sampling with a primary air flow calibrator. The personal air sampler was active for the whole time the farmers were spraying acetochlor. The XAD-2 and glass fiber filters were separated and frozen at −20 °C until analysis.

#### 2.1.2. Dermal Patch Sampling

**Figure 1:**
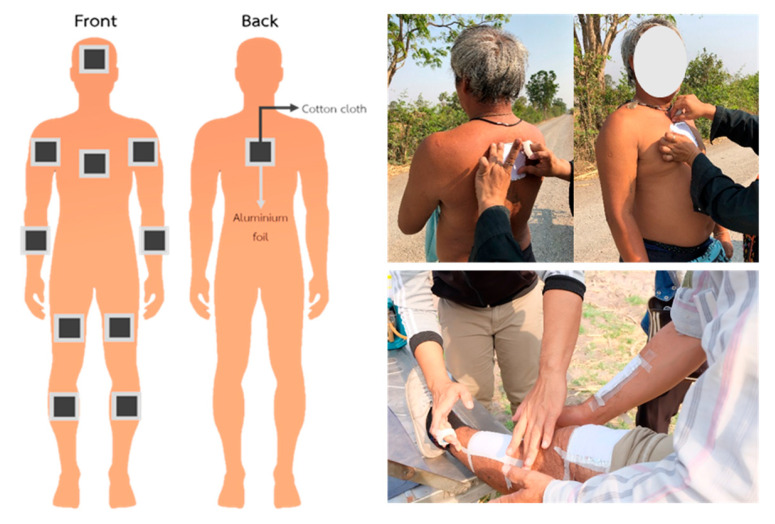
Patches were placed in different locations on bare skin.

Dermal contact samples were collected by using a cotton patch (10 cm × 10 cm) sewn on top of an aluminum foil pad that was taped in place on the bare skin of farmers before spraying. Patches were located in 11 positions, including one patch on the face, chest, and back and two patches on the upper arms (left and right), forearms (left and right), thighs (left and right), and lower legs (left and right) (Figure 1). The farmer’s typical clothing was worn over the dermal patch samplers. After spraying, the dermal patch samples were removed by forceps and the aluminum foil was removed. The dermal patch samples were stored in sealed bottles and were frozen at −20 °C until analysis.

### 2.2. Analysis of Personal Breathing Zone Air Samples and Dermal Patch Samples

#### 2.2.1. Chemical Reagents

Acetochlor and dimethachlor (internal standard for air sample analysis) were obtained from LGC Dr. Ehrenstorfer (Augsburg, Germany). Hexane and acetone were obtained from Merck (Darmstadt, Germany), and Acetochlor-ethane sulfonic acid (Acetochlor ESA) was obtained from LGC Dr. Ehrenstorfer (Augsburg, Germany). Additionally, 2,4,6-Trimethylaniline (internal standard for urine sample analysis) was obtained from Alfa Aesar (Lancashire, UK). Dichloromethane and 50% NaOH and HCl were obtained from Merck (Darmstadt, Germany).

#### 2.2.2. Analysis of Personal Breathing Zone Air Samples 

The analysis of air samples was carried out following the method for analysis of dermal patch samples by Mahaboonpeeti et al. [20]. The front and back section of XAD-2 and the glass fiber filter were separated into different tubes. Following this, 1 mL of hexane and acetone (1:1 *v*/*v*) were pipetted into the tubes, and, subsequently, the cap was closed and sealed with parafilm. Then, the samples were mixed and sonicated for 30 min. Next, 200 µL of the extracted solutions and 30 µL of dimethachlor as the internal standard were injected for gas chromatography-mass spectrometry (GC-MS). The GC-MS conditions were as follows: HP-5MS column (30 m length × 250 μm id × 0.25 µm film thickness), splitless injection mode, and inlet temperature of 250 °C. The temperature of the column was initiated at 50 °C for 1 min, then raised at 10 °C/min to 200 °C, followed by an increase at 3 °C/min to a final temperature of 230 °C for 4 min and post run at 250 °C for 2 min. The flow rate was set at 1 mL/min using helium as the carrier gas. The fixed electron energy was set at 70 eV relative to the standard autotune. The retention times of acetochlor and dimethachlor were 18.2 and 18.0 min, respectively. The quantitation ions were *m*/*z* 146 and *m*/*z* 223 for acetochlor and *m*/*z* 134 and *m*/*z* 197 for dimethachlor. Additionally, the calibration curve of acetochlor in the air sample was prepared at concentrations of 25, 50, 100, 300, 500, 1000, 1500, and 2000 ng. The recovery of acetochlor in air samples of 100 ng and 1000 ng ranged from 99.3 to 103.3% with a relative standard deviation (RSD) of less than 4%. The limit of detection (LOD) for acetochlor in the air samples was 1.7 ng.

#### 2.2.3. Analysis of Dermal Patch Samples

For dermal sampling, the cotton patch was put into a tube with 8 mL of hexane and acetone (1:1 *v*/*v*), and the cap was closed. Then, the sample was mixed and sonicated for 20 min. Subsequently, 300 µL of the extracted solution and 20 µL of the dimethachlor internal standard were injected into GC-MS using the same conditions implemented for the air sample analysis. Acetochlor concentrations of cotton patch samples were calculated following the US Environmental Protection Agency guidelines [21]. The length of spraying in hours (h) was used to estimate the dermal contact exposure in µg/h. The concentration of acetochlor on cotton patch samples was expressed as micrograms of acetochlor per square centimeter (100 cm^2^) of cotton pad per hour of exposure. After that, the dermal patch concentration (µg/cm^2^/h) was multiplied by the adult body surface areas [21] to obtain dermal contact exposure in µg/h. The estimated total dermal contact exposure was calculated by summing the µg/h levels for the 11 dermal pad samples taken on each individual. The calibration curve of acetochlor in the patch sample was prepared at concentrations of 0.2, 0.4, 0.8, 2.4, 4, 8, 12, and 16 µg. The recovery of acetochlor in patch samples of 0.8 µg and 8 µg ranged from 97.9 to 104.5% with RSD less than 4.5%. Lastly, the limit of detection (LOD) of acetochlor in the patch samples was 0.01 µg.

#### 2.2.4. Analysis of Urine Sample

**Figure 2:**
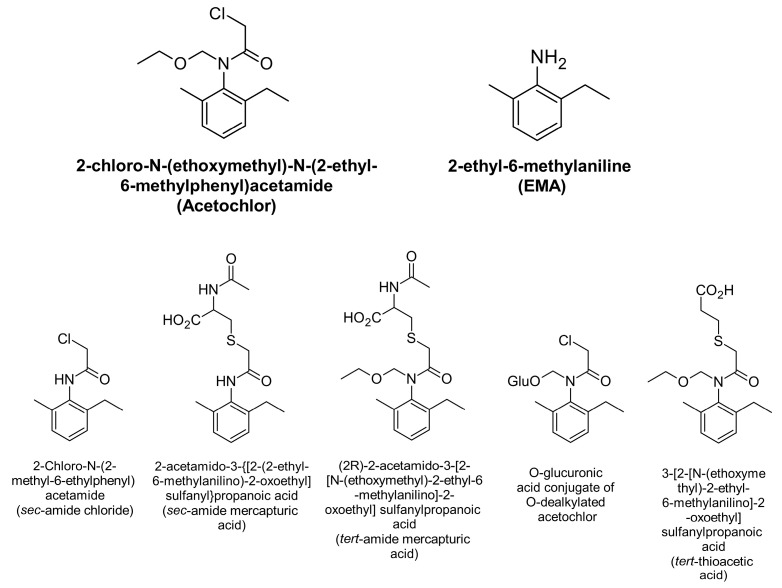
Structural formulae of acetochlor, acetochlor metabolites, and 2-ethyl-6-methylaniline (EMA).

In monkeys, three main metabolites are formed from acetochlor which are excreted in urine: tert-amide mercapturic acid (19.9–31.6%), O-glucuronic acid conjugate of O-dealkylated acetochlor (12.7–22.8%), and sec-amide mercapturic acid (more than 5%). There were also two minor metabolites identified: sec-amide chloride (3.9%) and tert-thioacetic acid (3.5%) [22]. The analysis of urinary acetochlor metabolites can be conducted by hydrolysis of the metabolites into 2-ethyl-6-methylaniline (EMA), which includes up to 70% of the acetochlor metabolites [22]. The structural formulae of acetochlor, acetochlor metabolites, and EMA are shown in Figure 2. The analysis method for 2-ethyl-6-methylaniline (EMA) was modified from Driskell et al. [23]. Urine samples (3 mL) were hydrolyzed following the Driskell method for 1.5 h. The mixture was neutralized, and 25 µL of 2,4,6-trimethylaniline was added as an internal standard. Then, 1.5 mL of dichloromethane was added to extract the 2-ethyl-6-methylaniline (EMA) by shaking for 5 min and centrifugation at 5000 rpm for 5 min. The extraction was performed twice. The solution was concentrated to 300 µL in a nitrogen evaporator because the 2-ethyl-6-methylaniline (EMA) is volatile and, therefore, concentration to dryness could be avoided using this method. The solution (300 µL) was injected into the GC-MS for analysis. The GC-MS conditions were as follows: DB-200 column (30 m length × 250 μm id × 0.25 µm film thickness), splitless injection mode, and the inlet temperature of 250 °C. The temperature of the column was initiated at 70 °C for 2 min and then raised at 20 °C/min to 100 °C, at 5 °C/min to 130 °C, and at 20 °C/min to a final temperature of 200 °C and post run at 250 °C for 2 min. The flow rate was set at 1 mL/min using helium as the carrier gas. The fixed electron energy was set at 70 eV relative to the standard autotune. The retention times of EMA and 2,4,6-trimethylaniline were 9.0 and 9.2 min, respectively. The quantitation ions were *m*/*z* 120 and *m*/*z* 135 for EMA and *m*/*z* 120, *m*/*z* 134, and *m*/*z* 135 for 2,4,6-trimethylaniline. In addition, the calibration curve for the EMA in urine used concentrations of 50, 250, 500, 1000, and 1500 ng/mL, and the recovery of EMA concentrations of 100 and 1000 ng/mL ranged from 94.5 to 101.2% with an RSD of less than 5%. The limit of detection (LOD) of EMA was 2 ng/mL. Moreover, all urinary metabolites were corrected for creatinine levels by dividing the metabolite concentration by the creatinine concentration, producing units of ng metabolite/g for the creatinine. Furthermore, the creatinine in urine was analyzed using an enzymatic method with a linear concentration range of 1–500 mg/dL and a detection limit of 0.16 mg/dL [24]. EMA concentrations were presented as µg/g creatinine, and the geometric mean (GM) and geometric standard deviation (GSD) were calculated in µg/g creatinine.

### 2.3. Statistical Analysis

All statistical analyses were conducted using SPSS for Windows, version 23 (IBM Thailand Co., Ltd., Bangkok, Thailand). A descriptive analysis of the demographic characteristics was carried out using the mean, standard deviation, and independent *t*-tests. The breathing zone air samples, dermal patch samples, and urinary EMA concentrations were skewed, so the natural logarithm of the concentration was used in all analyses. Furthermore, the geometric mean (GM) was calculated as the exponentiated mean of the natural log values, and the geometric standard deviation (GSD) was calculated as the exponentiated standard deviation of the natural log values. For concentrations below the detection limit, we substituted the detection limit by dividing the detection limit with the square root of 2 when the GSD was <3, and when the GSD was ≥3, the detection limit divided by two was used [25]. The natural log values for the urinary EMA concentrations at morning void for the day before spraying, end of spraying task, and the day after spraying were compared using a repeated measures ANOVA with post hoc Fisher’s LSD test. The urinary EMA concentrations between backpack and tractor sprayers were compared using the Mann–Whitney U test. The correlation between the natural logarithm of the acetochlor concentrations in the breathing zone air samples with those of total body dermal patch samples and urinary EMA concentration was analyzed using Pearson correlation.

## 3. Results

Based on the characteristics collected, the average age of the farmers was 49.7 years (Table 1). In addition, there were more male (75%) participants and most had graduated from high school or higher (51.7%). The majority were current alcohol drinkers (66.7%), though only 18.3% were smokers. Many participants also had a BMI that qualified as obese or overweight (45%), and the majority (76.7%) have been spraying pesticides for more than 10 years. Two types of spraying equipment were used, with 83.3% using backpack sprayers and 16.7% using tractor sprayers. The average time of spraying was 40.2 min and ranged from 20 to 120 min.

While spraying, all farmers wore long sleeve shirts (100%), 80% wore shoes, and 60–62% wrapped a cloth around their face when spraying. However, backpack sprayers were significantly more likely to wear long pants (98%) than tractor sprayers (80%). Few farmers also wore gloves, cotton masks, goggles, or boots (Table 2).

The detection frequency of urinary EMA concentrations was 66.7, 90.0, and 93.3% on the day before spraying, at the end of the spraying task, and on the day after spraying, respectively (Table 3). The urinary EMA concentrations at the end of spraying and on the day after spraying were significantly higher than on the day before spraying, but the EMA concentrations at the end of the spraying event and the day after spraying were not significantly different. Additionally, the urinary EMA concentrations between backpack and tractor sprayers were not significantly different on the day before spraying, the spraying day, and the day after spraying. The urinary EMA concentrations on the day after spraying were also not significantly different based on smoking or drinking status, gender or BMI category, nor were they significantly different between sprayers who sprayed only acetochlor and those who sprayed acetochlor mixed with other pesticides.

The acetochlor concentrations in the breathing zone air samples of backpack sprayers were not significantly different from those of tractor sprayers (Table 4). 

Moreover, for dermal patch samples, the acetochlor concentrations of tractor sprayers were not significantly different from those of backpack sprayers. However, the sprayed area, acetochlor solution used, and spraying duration of tractor sprayers were significantly higher in tractor sprayers than those of backpack sprayers.

The acetochlor concentrations in the breathing zone air samples were significantly correlated with those of the total body dermal patch samples and the urinary EMA concentrations after spraying (Table 5).

## 4. Discussion

The average age of farmers in this study was 49.7 years old, which was similar to the average age of 49.5 years in Bootsikeaw et al.’s [26] study in Thailand, and much higher than the 29.8 years average reported age in the study of Jallow et al. [27] in Kuwait, Asia or the 35.2 years reported in the study of Mergia et al. [28] in Ethiopia, Africa. This current study had only 25% female sprayers. It is a tough and difficult job for females to carry a backpack sprayer on their shoulders; however, there was no significant difference in exposures between male and female sprayers. Other studies have also found that men have more responsibility for planting and pesticide application than women [29]. In the Ethiopia study, more than 98% of small-scale farmers were men because farm activities and pesticide spraying were primarily performed by men [28]. In Kuwait, farm activities, especially pesticide use, were performed exclusively by men [27]. Additionally, in this current study, a larger fraction of our farmers had sprayed pesticides for more than 10 years (76.7%) compared to 10.4% in the Ethiopian study and 41.9% in the Kuwait study [26,27]. The sprayers were also less likely to smoke cigarettes (18.3%) than the average Thai population (22.2%) [30], but the percentage of sprayers who reported drinking alcohol (66.7%) was similar to the occasional or regular drinkers (65%) reported in the general Thai population [31].

Moreover, this study found that the percentage of backpack sprayers wearing PPE (primarily long sleeve shirts, long pants, shoes, and face cloths) was higher than those reported by Mergia et al. [28] and Mahaboonpeeti et al. [20]. However, it has been reported that farmers do not have sufficient knowledge of pesticide toxicity and how to use pesticides safely [27].

This study selected 2-ethyl-6-methylaniline (EMA) as the acetochlor metabolite to estimate acetochlor exposure in spraying farmers. EMA is the hydrolysis product of the five identified metabolites of acetochlor: tert-amide mercapturic acid, O-glucuronic acid conjugate of O-dealkylated acetochlor, sec-amide mercapturic acid, sec-amide chloride, and tert-thioacetic acid, which makes up approximately 59.5% to 70.2% of all acetochlor metabolites [22]. Barr et al. [32] studied acetochlor mercapturate (ACM) and EMA, and proposed another new biomarker. In this study, we selected subjects who sprayed only acetochlor (61.7%) and acetochlor mixed with other chemicals, such as paraquat, 2,4-dichlorophenoxyacetic acid, cypermethrin, ametryn, chlorpyrifos, and diuron (38.3%). The comparison of EMA concentrations between subjects who sprayed pure acetochlor and acetochlor mixed with other chemicals was not significantly different. The sprayers in this study did not use metolachlor or chloroacetanilide herbicides, which could be changed to EMA after the hydrolysis process [32]. The detection frequency of urinary EMA was 66.7, 90.0, and 93.3% on the day before spraying, at the end of the spraying task and the day after spraying, respectively. On the day before spraying, urinary EMA concentrations were detected in only 66.7% farmers because the farmers might not have used acetochlor for a while before our visit, but it was still measurable in the urine, perhaps due to field contamination or storage near the home. Gustin et al. [33] reported the detection frequency of urinary EMA (24 h composite urine) of farmers spraying acetochlor as 15, 100, and 100% the day before spraying, the spraying day, and the day after spraying using open- and closed-tractor cab spraying. Furthermore, the detection frequency of Gustin et al.’s [33] study was higher than the current study on the spray days because of the longer duration of spraying, higher spraying volume, and spraying continuously for 5 days. Gustin et al.’s [33] study also reported urinary EMA concentrations of open- and closed-tractor cabins of 0.004 and 0.002 mg/kg bw/day, respectively. Moreover, the Curwin et al. study in the US reported a GM of urinary ACM of 8.0 µg/L in four sprayers [34]. The GM of urinary EMA on the day after spraying in this current study was 111.0 µg/g creatinine (947.4 nmole/L) ranging from 1.3 to 1865.3 µg/g creatinine (10.6–10,202.5 nmole/L). Barr et al. [32] reported the range of urinary EMA of 7.2–1805 nmole/L for seven sprayers, where urine samples were collected within 24–48 h of acetochlor application. The levels in the Barr et al. study were likely lower because the applicators collected a 24 h composite urine sample and had closed cabin tractors with air conditioning and dust and charcoal filters [32,35,36]. In this study, the urinary EMA on the day after application was probably the highest because the body had to metabolize the exposure, and the half-life of acetochlor in urine was reported to be 1.2 days in monkeys [33]. 

The acetochlor concentrations in the breathing zone air samples of backpack sprayers were slightly higher than tractor sprayers but not significantly different. Since the vapor pressure of acetochlor is low (1.67 × 10^−7^ mm Hg at 20 °C), it is present more as a particle than vapor. Currently, there is no exposure limit for acetochlor, although it is widely used for agricultural work in many countries, such as the United States [11], China [13], and Thailand [7]. An airborne exposure limit for acetochlor should be established for workers’ health and safety in the workplace.

**Figure 3:**
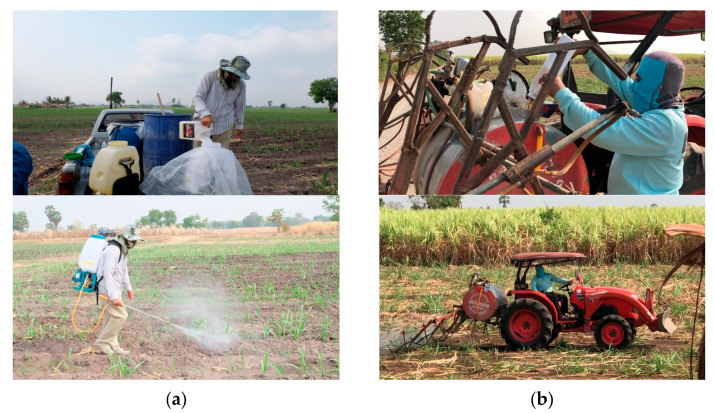
The backpack and tractor sprayers while mixing and spraying acetochlor. (**a**) The backpack sprayer; (**b**) the tractor sprayer.

Regarding acetochlor on the dermal patches, the acetochlor concentrations of tractor sprayers were not significantly different from those of backpack sprayers. Since the acetochlor concentrations in the breathing zone between the tractor and backpack sprayers were not significantly different, it is possible that contamination occurred during the period of mixing acetochlor with water and loading it into the tractor tank. We found that tractor sprayers load the acetochlor solution at the height of the farmer’s head and chest, which could lead to spilling and splashing of the acetochlor solution onto the head and chest (Figure 3). We also noted that the forehead and chest patch samples for tractor sprayers were higher than those of backpack sprayers. 

Like the air concentration, the acetochlor concentration on the back, arms, legs, and total body patch samples was not significantly different between the backpack sprayers and the tractor sprayers. The factors affecting exposures between the backpack and tractor sprayers included the area of the field being sprayed onto, the amount of acetochlor solution used, and the spraying duration, as well as other likely factors, such as nozzle size, spraying pressure, application maintenance, application height, and the angle of spraying [17]. Additionally, the backpack sprayers were more easily exposed to acetochlor than tractor sprayers since the sprayer points the nozzle at the soil close to their feet. However, the tractor sprayers used a larger amount of acetochlor solution and sprayed a larger area for a longer period of time than the backpack sprayers. Because both groups mostly wore similar clothing (long pants and long sleeve shirts and a cloth around the face), the closer proximity and smaller volumes of spraying may have resulted in equivalent exposures (µg/h) compared to the larger volume and downwind spray of the tractor drivers. 

The back, arms, and leg patches were the most exposed body areas to acetochlor; these results are similar to Mahaboonpeeti et al. [20] and Bootsikeaw et al. [26]. Regarding the backpack sprayers, their back exposure to acetochlor (2.8–10,472.8 µg/h) could occur due to acetochlor leakage of an old sprayer tank during spraying. Arm exposure to acetochlor (4.5–4368.1 µg/h) could also be caused by spilling of the acetochlor solution from the tank during loading and mixing or blowback of the spray while applying. Lastly, high leg concentrations of acetochlor (10.5–32,403.7 µg/h) are most likely caused by the position and angle of the nozzle used to spray on the ground. Even though 98% of the backpack and 80% of tractor sprayers wore long pants and 100% wore long sleeve shirts, they were still exposed to acetochlor at high concentrations.

The urinary EMA concentrations of backpack sprayers on the day after spraying were slightly higher than that of the tractor sprayers but not significantly different. However, Gustin et al. [33] showed that the open-cabin applicators had significantly higher EMA concentrations compared to the closed-cabin applicators. However, in this study, the tractor sprayers were not normally in a closed cabin. 

The acetochlor concentrations of the breathing zone air samples were significantly correlated with those of the total body dermal patch samples and the urinary EMA concentrations after spraying (Table 5). However, the acetochlor concentrations of the total body dermal patch samples were not significantly correlated with urinary EMA concentrations after spraying. As the skin is an effective barrier, it may take a longer time to pass through it and, thus, the amount of acetochlor entering the bloodstream is limited. 

## 5. Conclusions

For backpack and tractor sprayers, exposure to the herbicide acetochlor was not significantly different for breathing zone air samples, total body dermal samples, and urinary EMA concentrations on the day after spraying. In addition, the urinary EMA concentrations on the day before spraying were significantly lower than those at the end of the spraying task as well as the day after spraying (which had the highest levels). The two groups of sprayers also used only regular clothing for PPE, resulting in inhalation and dermal exposures that increased their EMA levels when they sprayed. Furthermore, the breathing zone air samples were significantly correlated with those of the levels of the total body dermal patch samples and with the urinary EMA concentrations after spraying. Thus, both air and dermal exposure contribute to urinary EMA levels, and personal protective equipment improvements are needed. Future education of farmers should focus on the need to wear improved PPE, such as plastic aprons, coveralls, and rubber gloves during mixing and spraying. 

## Figures and Tables

**Table 1:** Characteristics of farmers (*n* = 60).

Variables		Total (*n*)	Conventional Farmers *n* (%)
Age			
	Min–max	60	18–69
	Mean (SD)		49.7 (12.9)
Sex			
	Male	45	45 (75.0)
	Female	15	15 (25.0)
Educational level			
	Elementary	29	29 (48.3)
	High school or higher	31	30 (51.7)
Marital status			
	Single	7	7 (11.7)
	Married	51	51 (85.0)
	Widowed/divorced	2	2 (3.3)
Alcohol intake			
	Current drinker	40	40 (66.7)
	Nondrinker	20	20 (33.3)
Smoking			
	Current smoker	11	11 (18.3)
	Nonsmoker	49	49 (81.7)
BMI (kg/m^2^)			
	Underweight (<18.5)	7	7 (11.7)
	Healthy Weight (18.5–<25.00)	26	26 (43.3)
	Overweight (25.00–<30.00)	19	19 (31.7)
	Obesity (30.0 or higher)	8	8 (13.3)
Spraying pesticides in agricultural fields (years)			
	≤10	14	14 (23.3)
	>10	46	46 (76.7)
Spraying equipment			
	Backpack sprayers	50	50 (83.3)
	Tractor sprayers	10	10 (16.7)

**Table 2:** Personal protective equipment (PPE) used while spraying (*n* = 60).

PPE	Backpack (*n* = 50) *n* (%)	Tractor (*n* = 10) *n* (%)
Goggles	2 (4)	0
Cloth gloves	12 (24)	0
Latex gloves	2 (4)	1 (10)
Long sleeve shirts	50 (100)	10 (100)
Shoes	40 (80)	8 (80)
Boots	6 (12)	0
Socks	7 (14)	0
Short pants	1 (2)	2 (20)
Long pants	49 (98)	8 (80)
Large brim straw hat	16 (32)	1 (10)
Cloth wrapped around face	31 (62)	6 (60)
Cotton mask	3 (6)	2 (20)
Balaclava	11 (22)	3 (30)

**Table 3:** Comparison of the natural log of EMA concentrations at different times of urine collection (µg/g creatinine) using repeated measures ANOVA with post hoc Fisher’s LSD test and comparison of EMA concentrations at different times between backpack and tractor sprayers using the Mann–Whitney U test on the natural log of EMA.

Urine Collection		EMA (µg/g Creatinine)			Comparison of EMA between Backpack vs. Tractor ^1^
		Total Farmers (*n* = 60)	Backpack (*n* = 50)	Tractor (*n* = 10)	
The day before spraying					
	Detection frequency (%)	40 (66.7)	31 (62.0)	9 (90.0)	
	GM (GSD) ^2^	11.5 (8.6)	10.2 (9.2)	20.7 (5.7)	*p* = 0.242
	Range (Min–Max)	0.6–1099.0	0.6–1099.0	1.4–308.6	
The end of spraying task					
	Detection frequency (%)	54 (90.0)	44 (88.0)	10 (100.0)	
	GM (GSD) ^2^	88.5 (8.6)	84.0 (10.0)	115.1 (3.2)	*p* = 0.937
	Range (Min–Max)	1.2–3849.8	1.2–3849.8	11.4–1155.6	
The day after spraying					
	Detection frequency (%)	56 (93.3)	46 (92.0)	10 (100.0)	
	GM (GSD) ^2^	111.0 (6.4)	117.3 (6.6)	84.3 (5.8)	*p* = 0.579
	Range (Min–Max)	1.3–1865.3	1.3–1865.3	4.6–823.4	
Comparison of total farmers’ EMA ^3^					
The day before spraying and the end of spraying task				*p* < 0.001 *	
The day before and after spraying				*p* < 0.001 *	
The end of spraying task and the day after spraying				*p* = 0.350	

^1^ Mann–Whitney U test on natural log of EMA; ^2^ GM = geometric mean, GSD = geometric standard deviation; ^3^ Repeated measures ANOVA with post hoc Fisher’s LSD test on natural log of EMA; * *p* < 0.05.

**Table 4:** Comparison on acetochlor air concentrations, dermal patch levels, and spraying factors between backpack and tractor spraying.

Acetochlor/EMA Concentrations		Backpack (*n* = 50)	Tractor (*n* = 10)	*p*-Value
Breathing zone air sample (µg/m^3^)				0.403
	Detection frequency (%)	50 (100.0)	10 (100.0)	
	GM (GSD) ^1^	13.7 (3.4)	9.3 (6.1)	
	Range (Min–Max)	0.3–92.2	0.1–83.9	
Dermal patch sample (µg/h)				
Forehead				0.051
	Detection frequency (%)	50 (100.0)	10 (100.0)	
	GM (GSD) ^1^	5.3 (3.5)	12.9 (3.9)	
	Range (Min–Max)	0.8–1398.7	1.2–105.2	
Chest				0.052
	Detection frequency (%)	50 (100.0)	10 (100.0)	
	GM (GSD) ^1^	24.1 (2.8)	52.6 (5.1)	
	Range (Min–Max)	3.4–1358.8	6.4–3353.0	
Back				0.814
	Detection frequency (%)	50 (100.0)	10 (100.0)	
	GM (GSD) ^1^	41.0 (6.3)	35.2 (7.6)	
	Range (Min–Max)	2.8–10,472.8	5.9–9945.2	
Arms				0.400
	Detection frequency (%)	50 (100.0)	10 (100.0)	
	GM (GSD) ^1^	33.4 (2.9)	45.6 (2.8)	
	Range (Min–Max)	4.5–4368.1	19.0–697.1	
Legs				0.798
	Detection frequency (%)	50 (100.0)	10 (100.0)	
	GM (GSD) ^1^	201.8 (4.7)	231.6 (4.4)	
	Range (Min–Max)	10.5–32,403.7	36.9–6309.8	
Total body				0.884
	Detection frequency (%)	50 (100.0)	10 (100.0)	
	GM (GSD) ^1^	503.0 (4.5)	466.0 (4.6)	
	Range (Min–Max)	24.7–37,645.8	75.6–19,788.2	
Spraying information				
Sprayed area (Ha)				<0.001 *
	GM (GSD) ^1^	0.5 (1.8)	1.6 (1.7)	
	Range (Min–Max)	0.2–3.0	0.5–2.4	
Acetochlor solution used (L)				<0.001 *
	GM (GSD) ^1^	83.4 (1.5)	823.8 (1.5)	
	Range (Min–Max)	40–200	300–1000	
Spraying duration (min)				0.027 *
	GM (GSD) ^1^	35.5 (1.4)	45.1 (1.8)	
	Range (Min–Max)	20–120	25–120	

*p*-values were calculated by using independent *t*-Test. * *p* < 0.05; ^1^ GM = geometric mean, GSD = geometric standard deviation.

**Table 5:** The relationship between acetochlor concentrations in the breathing zone air samples, total body dermal patch levels, and EMA concentrations in urine after spraying using Pearson correlation (*n* = 60). Correlations were performed on the natural log of all metrics.

Acetochlor/EMA Concentrations		Correlation	*p*-Value
Breathing zone air sample (µg/m^3^)			
	Total body dermal patch samples (µg/h)	0.269	0.037 *
	Urinary EMA concentrations the day after spraying (µg/g creatinine)	0.381	0.003 *
Total body dermal patch samples (µg/h)			
	Urinary EMA concentrations the day after spraying (µg/g creatinine)	0.022	0.867

* *p* < 0.05.

## Data Availability

The data presented in this study are available on request from the corresponding author.

## References

[1] National Statistical Office Statistics of Land, Whole Kingdom: 2012–2022. http://statbbi.nso.go.th/staticreport/Page/sector/TH/report/sector_11_18_TH_.xls.

[2] National Statistical Office (2022). The Informal Employment Survey 2022. http://www.nso.go.th/sites/2014/Pages/สำรวจ/ด้านสังคม/แรงงาน/แรงงานนอกระบบ.aspx.

[3] Bureau of Occupational and Environmental Diseases, Ministry of Public Health Report Occupational and Environmental Diseases and Health Hazards in 2017. https://ddc.moph.go.th/uploads/ckeditor2//files/01_envocc_situation_60.pdf.

[4] Office of Agricultural Economics Quantity and Value of Imports of Agricultural Pesticides 2018–2022. http://www.oae.go.th/view/1/ปัจจัยการผลิต/TH-TH.

[5] Heydens W.F., Lamb I.C., Wilson A.G. (2010). Chloracetanilides. Hayes’ Handbook of Pesticide Toxicology.

[6] Environmental Protection Agency (1994). Federal Register Office of the Federal Register.

[7] Department of Agriculture Report The Herbicide Imported in 2007–2021. https://data.go.th/dataset/importherbicidevol.

[8] Office of the Cane and Sugar Board Sugarcane Area Report 2021–2022. http://www.ocsb.go.th/upload/journal/fileupload/13813-1585.pdf.

[9] Ashby J., Kier L., Wilson A., Green T., Lefevre P., Tinwell H. (1996). Evaluation of the potential carcinogenicity and genetic toxicity to humans of the herbicide acetochlor. Hum. Exp. Toxicol..

[10] Dearfield K.L., McCarroll N.E., Protzel A., Stack H.F., Jackson M.A., Waters M.D. (1999). A survey of EPA/OPP and open literature on selected pesticide chemicals: II. Mutagenicity and carcinogenicity of selected chloroacetanilides and related compounds. Mutat. Res. Genet. Toxicol. Environ. Mutagen..

[11] Lerro C.C., Koutros S., Andreotti G., Hines C.J., Blair A., Lubin J. (2015). Use of acetochlor and cancer incidence in the Agricultural Health Study. Int. J. Cancer.

[12] Crump D., Werry K., Veldhoen N., Van A.G., Helbing C.C. (2002). Exposure to the herbicide acetochlor alters thyroid hormone-dependent gene expression and metamorphosis in Xenopus Laevis. Environ. Health Perspect..

[13] Li L., Wang M., Chen S., Zhao W., Zhao Y., Wang X. (2016). A urinary metabonomics analysis of long-term effect of acetochlor exposure on rats by ultra-performance liquid chromatography/mass spectrometry. Pestic. Biochem. Physiol..

[14] European Food Safety Authority (2011). Conclusion on the peer review of the pesticide risk assessment of the active substance acetochlor. EFSA J..

[15] Das N., Maske N., Khawas V., Chaudhary S., Dhete R. (2015). Agricultural fertilizers and pesticides sprayers—A review. Int. J. Innov. Res. Sci. Technol..

[16] Bhatkar A.K., Khope P., Chaudhari P. (2016). A Review: Development of Pesticide Spraying Machine. IJRET Int. J. Res. Eng. Technol..

[17] Franke A., Kempenaar C., Holterman H., Van Z.J. (2010). Spray Drift from Knapsack Sprayers: A Study Conducted within the Framework of the Sino-Dutch Pesticide Environmental Risk Assessment Project PERAP.

[18] Kongtip P., Nankongnab N., Pundee R., Kallayanatham N., Pengpumkiat S., Chungcharoen J., Phommalachai C., Choochouy N., Sowanthip P., Khangkhun P. (2021). Acute changes in thyroid hormone levels among Thai pesticide sprayers. Toxics.

[19] NIOSH Manual of Analytical Methods (1994). Chlorinated and Organonitrogen Herbicides: Method 5602 (Air Sampling).

[20] Mahaboonpeeti R., Kongtip P., Nankongnab N., Tipayamongkholgul M., Bunngamchairat A., Yoosook W. (2018). Evaluation of dermal exposure to the herbicide alachlor among vegetable farmers in Thailand. Ann. Work Expo. Health.

[21] United States Environmental Protection Agency Occupational and Residential Exposure Test Guidelines: OPPTS 875.2400 Dermal Exposure. https://www.regulations.gov/document?D=EPAHQ-OPPT-2009-0157-0012.

[22] Shah P., Moretto A. Acetochlor. Proceedings of the Pesticide Residues in Food 2015, Joint FAO/WHO Meeting on Pesticide Residues.

[23] Driskell W., Hill R., Shealy D., Hull R., Hines C. (1996). Identification of a major human urinary metabolite of alachlor by LC–MS/MS. Bull. Environ. Contam. Toxicol..

[24] Beckmann Coulter, Instruction for Use. Creatinine. https://www.beckmancoulter.com/wsrportal/techdocs?docname=/cis/A69463/%%/EN.

[25] Hornung R.W., Reed L.D. (1990). Estimation of average concentration in the presence of nondetectable values. Appl. Occup. Environ. Hyg..

[26] Bootsikeaw S., Kongtip P., Nankongnab N., Chantanakul S., Sujirarat D., Mahaboonpeeti R. (2021). Urinary glyphosate biomonitoring of sprayers in vegetable farm in Thailand. Hum. Ecol. Risk Assess. Int. J..

[27] Jallow M.F., Awadh D.G., Albaho M.S., Devi V.Y., Thomas B.M. (2017). Pesticide knowledge and safety practices among farm workers in Kuwait: Results of a survey. Int. J. Environ. Res. Public Health.

[28] Mergia M.T., Weldemariam E.D., Eklo O.M., Yimer G.T. (2021). Small-scale farmer pesticide knowledge and practice and impacts on the environment and human health in Ethiopia. J. Health Pollut..

[29] Wang W., Jin J., He R., Gong H. (2017). Gender differences in pesticide use knowledge, risk awareness and practices in Chinese farmers. Sci. Total Environ..

[30] Jitnarin N., Kosulwat V., Rojroongwasinkul N., Boonpraderm A., Haddock C.K., Poston W.S. (2011). Socioeconomic status and smoking among thai adults: Results of the National Thai Food Consumption Survey. Asia Pac. J. Public Health.

[31] Wakabayashi M., McKetin R., Banwell C., Yiengprugsawan V., Kelly M., Seubsman S. (2015). Alcohol consumption patterns in Thailand and their relationship with non-communicable disease. BMC Public Health.

[32] Barr D.B., Hines C.J., Olsson A.O., Deddens J.A., Bravo R., Striley C.A. (2007). Identification of human urinary metabolites of acetochlor in exposed herbicide applicators by high-performance liquid chromatography-tandem mass spectrometry. J. Expo. Sci. Environ. Epidemiol..

[33] Gustin C.A., Moran S.J., Fuhrman J.D., Kurtzweil M.L., Kronenberg J.M., Gustafson D.I. (2005). Applicator exposure to acetochlor based on biomonitoring. Regul. Toxicol. Pharmacol..

[34] Curwin B.D., Hein M.J., Sanderson W.T., Barr D.B., Heederik D., Reynolds S.J. (2005). Urinary and hand wipe pesticide levels among farmers and nonfarmers in Iowa. J. Expo. Sci. Environ. Epidemiol..

[35] Hines C.J., Deddens J.A., Tucker S.P., Hornung R.W. (2001). Distributions and determinants of pre-emergent herbicide exposures among custom applicators. Ann. Occup. Hyg..

[36] Hines C.J., Deddens J.A., Striley C.A., Biagini R.E., Shoemaker D.A., Brown K.K., MacKenzie B.A., Hull R.D. (2003). Biological monitoring for selected herbicide biomarkers in the urine of exposed custom applicators: Application of mixed-effect models. Ann. Occup. Hyg..

